# Synaptonemal complex protein 2 (SYCP2) mediates the association of the centromere with the synaptonemal complex

**DOI:** 10.1007/s13238-016-0354-6

**Published:** 2017-02-01

**Authors:** Jianrong Feng, Shijuan Fu, Xuan Cao, Hao Wu, Jing Lu, Ming Zeng, Lin Liu, Xue Yang, Yuequan Shen

**Affiliations:** 1 State Key Laboratory of Medicinal Chemical Biology, Nankai University, Tianjin, 300071 China; 2 College of Life Sciences, Nankai University, Tianjin, 300071 China; 3 Synergetic Innovation Center of Chemical Science and Engineering, Tianjin, 300071 China

## Dear Editor,

In nearly all diploid eukaryotes, meiosis is the key phase in sexual reproduction. The typical process is completed by a round of DNA replication followed by two consecutive rounds of chromosome cell division (Ma et al., 2014; Page and Hawley, 2004). Homologous chromosome pairing and segregation occur in the first round of meiosis and is unique to mitosis. The second round of meiosis bears a similarity to the separation of sister chromatids. These events are crucial to eukaryotic fertility and are evolutionarily conserved from yeast to humans (Page and Hawley, 2004). A new individual phenotype is often different from that of the parents. Variation and diversity are mediated by the synapsis of homologous chromosomes and by genetic recombination. The mechanism of homology search and alignment remain elusive. One hallmark of the first round of meiosis is the assembly and disassembly of the synaptonemal complex (SC) (Ma et al., 2014). Some studies have shown that the SC plays an indispensable role in crossover formation and genetic exchange. If the SC is disrupted, the meiosis process becomes corrupted, leading to infertility, and even termination of the embryo (Kouznetsova et al., 2011). Down syndrome is a clinical example of defective SC formation (Bolor et al., 2009).

The SC was discovered more than half a century ago. Its structure, function and assembly mechanisms have been studied extensively using genetic, molecular and molecular phylogenetic analyses. SCs are composed of meiosis-specific proteins and are initiated in the early stage of meiotic prophase I (MI). SCs are fully assembled during the pachytene, one of the five MI sub-stages (leptotene, zygotene, pachytene, diplotene and diakinesis) (Fraune et al., 2012). Studies using electron microscopy demonstrated that functional SCs share a typical tripartite structure that is evolutionarily conserved across metazoan animals. The SC structure is composed of two lateral elements (LEs) that connect sister chromatids. Transverse filaments (TFs) provide a link between the LEs and central elements (CEs), generating a ladder-like or zipper-like structure (Page and Hawley, 2004). In recent years, studies of meiosis-associated proteins have provided new insight into the functional SC structure. In mammals, SYCE1, SYCE2, SYCE3 and TEX12 proteins have been identified as CEs. The first piece of SYCE3 structural information provided insight into understanding CE assembly (Lu et al., 2014). In TFs, the fibrillary molecular SYCP1 is the key component. Its long coiled coil domain belongs to one member of the inter-filament family. Knockout of the mouse TF gene SYCP1 led to apoptosis in most meiotic cells during the pachytene stage and subsequently led to both male and female infertility. SYCP2 and SYCP3 are the main component proteins of the LEs (Hosoya et al., 2012; Yang et al., 2006). The most recent structural evidence demonstrated that SYCP3 formed a tetramer, the N-terminal region bound DNA and the core domain assembled itself into the LEs (Syrjanen et al., 2014).

SYCP2 has 1,500 amino acids and contains multiple potential DNA binding motifs. Its C-terminal coiled coil region (CCR) interacts with SYCP3 (Winkel et al., 2009). In wild type mice, SYCP3 localized well to the SC, but in *sycp2* ^−/−^ mice, SYCP3 failed to localize to the LEs and accumulated as aggregates in the nucleus. Therefore, SYCP2 is essential for the incorporation of SYCP3 into the LEs (Yang et al., 2006). Disruption of SYCP2 in spermatocytes resulted in failed chromosomal synapsis. TUNEL assay and electron microscopy data showed that spermatocytes of *sycp2* ^−/−^ underwent apoptosis (Yang et al., 2006). The CCR has also been shown to interact with the SYCP3 and SYCP1 N-terminal regions in yeast two-hybrid screening and immunoprecipitation (Winkel et al., 2009; Yang et al., 2006). However, the functional role of the SYCP2 N-terminal region (NTR) remains unknown. In this study, we investigated the structure and function of the SYCP2 NTR. Unexpectedly, we discovered that the SYCP2 NTR associates with the centromere region.

**Figure 1:**
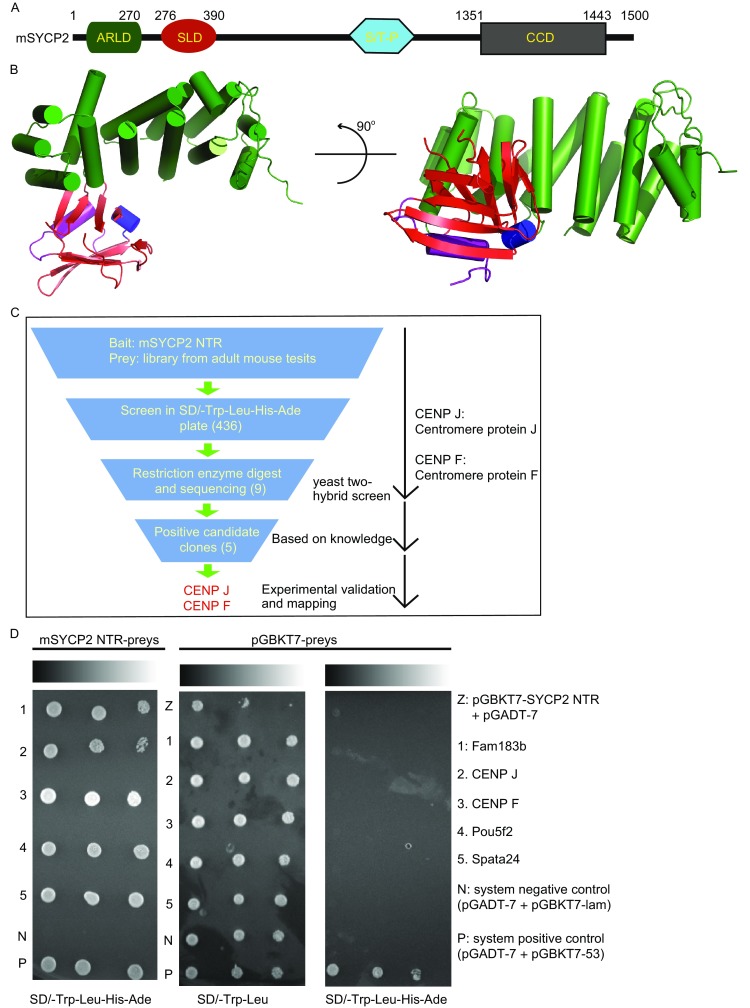
Structure of the mouse SYCP2 NTR. (A) Schematic representation of full-length mouse SYCP2 (1–1500 aa). (B) Illustrated representation of the mSYCP2 NTR structure. One molecule contains two subdomains: an ARLD and a SLD colored in green and red, respectively. (C) Search procedure for partners associated with the mSYCP2 NTR using a yeast two-hybrid system. A total of 436 candidates were preliminarily screened. (D) Verification using yeast two-hybrid screening with mSYCP2 NTR as the bait. Five potential candidates (1–5) were spotted onto SD/-Trp-Leu-His-Ade (QD). The corresponding bait with empty pGBKT7 vector was used as the negative control and spotted onto SD/-Trp-Leu and QD. Line Z (pGBKT7-NTR and empty pGADT7) determined auto-activation effects of the bait. Ten-fold serial dilutions of single AH109 colonies spotted on different nutrition-restricted plates. The co-transfected strains grew on QD medium for 3 days at 30°C

To crystallize mouse SYCP2 (mSYCP2), a series of constructs were screened. One construct of the mSYCP2 NTR (1–390 aa) was successfully crystallized, and the corresponding structure was determined with 2.1 Å resolution using the single anomalous dispersion method of the Selenium atom (Fig. 1A and 1B). The asymmetric unit contains one mSYCP2 NTR molecule. Of the total 390 amino acids, residues 1–114, 119–183, 193–319, and 329–390 were clearly visible in the electron density map. The missing regions were not built in the final model owing to poor electron density.

The overall structure is shaped like the letter “L” and contains two separate subdomains: an ARLD (armadillo-repeat-like domain) and an SLD (Spt16M-like domain). The ARLD is composed of fourteen helices (α1 to α14, Fig. S1) that may provide a platform for interactions with other partners. The SLD consists of a twisted ten-stranded β-sheet (β1 to β10) flanked by two helices (α15 to α16). Computational and structural homolog search analysis revealed that the ARLD structure is highly similar to that of the “required for cell differentiation (RCD-1)” protein (Fig. S2A; DALI Z-score of 13.3; r.m.s.d. of 3.9 Å). We also performed a DALI search for the SLD. Overall, the SLD structure is highly similar to the middle domain of the histone chaperone FACT (Fig. S2B; DALI Z-score of 10.6; r.m.s.d. of 2.6 Å). The Spt16M domain structure adopts a canonical Pleckstrin Homology (PH) domain architecture, which comprises a seven-stranded antiparallel-barrel that buries a hydrophobic core and has one end capped by a C-terminal helix. We speculate that the SLD may participate in a chromatin related process.

To further address the functional role of mSYCP2 NTR, yeast two-hybrid screening assays were performed using the mSYCP2 NTR as bait, and a matched cDNA library prepared from adult mouse testis was used as the prey. Following the standard procedure, all clones were selected using nutrition-restricted plates. Fifteen mmol/L 3-amino-1,2,4-triazole (3-AT) was added to the growth medium each time to inhibit low levels of His expression in a leaky manner when a potential candidate was validated.

In total, 9 prey clones were preliminarily captured by library screens. They all grew in SD/-Trp-Leu-His-Ade solid media, with the mSYCP2 NTR as the bait. To further validate the above candidates, an individual clone was co-transfected with an mSYCP2 NTR bait vector into the AH109 strain. Eventually, only five candidates that showed no apparent auto-activation effect (Fig. 1C) remained. After sequencing, the results were submitted to the VecScreen SERVER (http://www.ncbi.nlm.nih.gov/tools/vecscreen/), and then database searches were performed using the BLAST Service (NCBI, National Center for Biotechnology Service) (http://www.ncbi.nlm.nih.gov/BLAST). The identified proteins included two centromere components, CENP J and CENP F, meiosis specific function unknown protein Fam183b, transcription factor Pou5f2 and spermatogenesis associated 24 (Spata24) (Fig. 1D). The primary mSYCP2 partner candidate should be related to cell division, should be involved in meiosis and should co-localize with the SYCP2 axial element component. Implementing these criteria, proteins CENP J and CENP F were selected for further functional studies.

**Figure 2:**
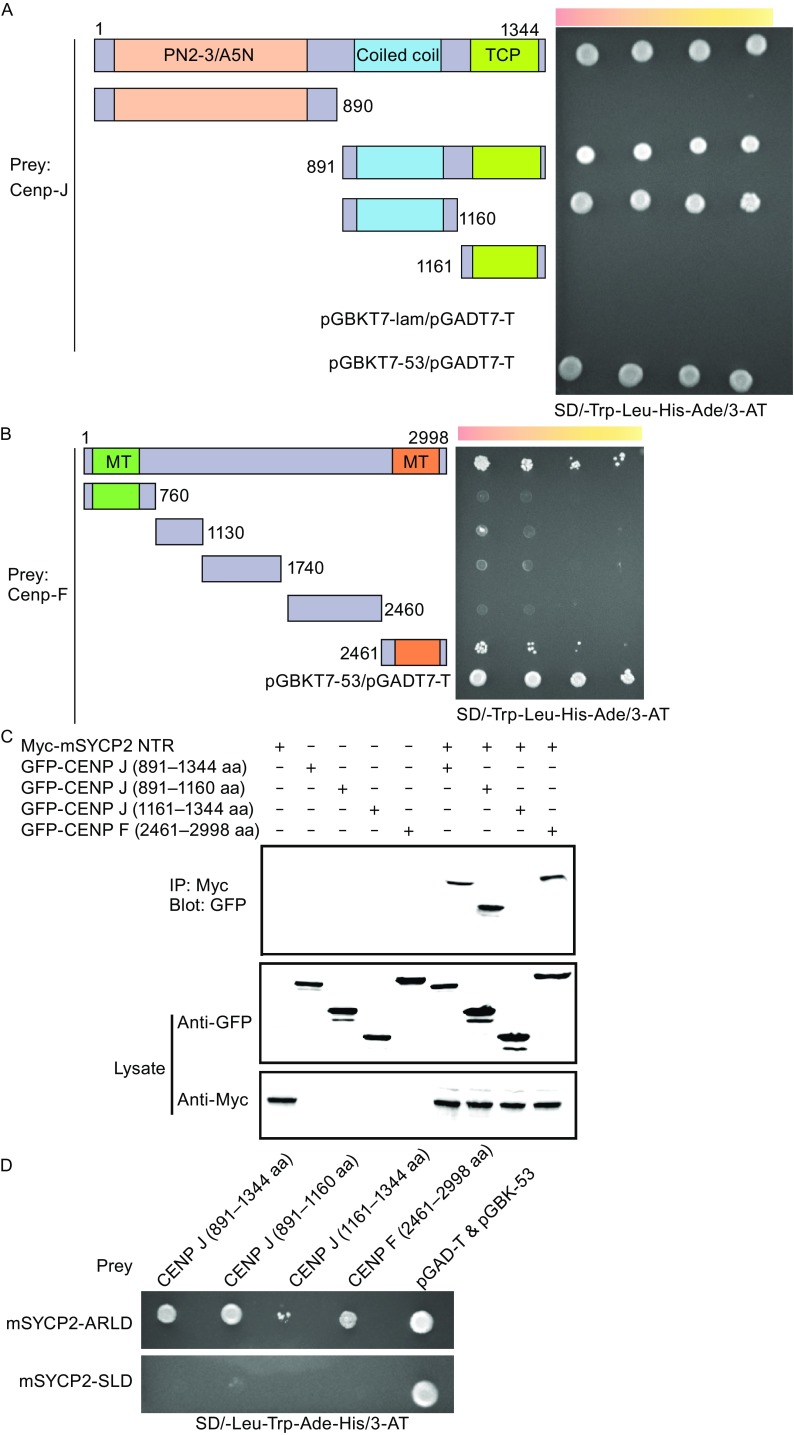

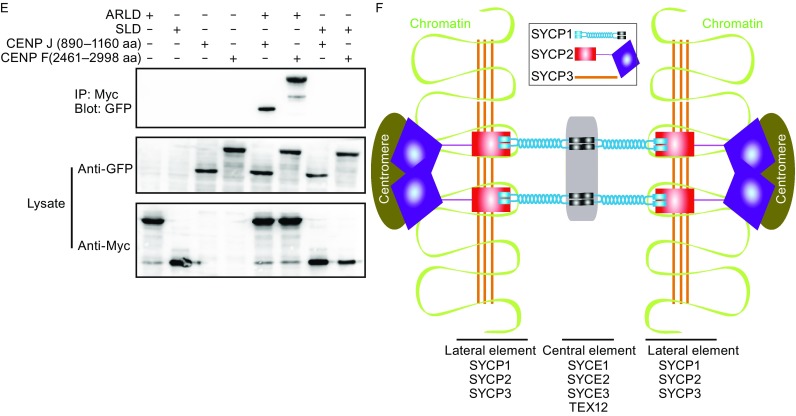
SC association with the centromere. (A) Yeast two-hybrid assay of CENP J domains associated with mSYCP2 NTR. The p53-T antigen pair and lam-T served as a system positive and negative control, respectively. The following bait constructs were used: CENP J wild type (1–1344 aa), CENP J (1–890 aa), CENP J (891–1344 aa), CENP J (891–1160 aa) and CENP J (1161–1344 aa). mSYCP2 NTR (1–390 aa) was used as the prey in this assay. (B) Yeast two-hybrid assay of CENP F domains with mSYCP2 NTR. The following bait constructs were used: CENP F wild type (1–2998 aa), CENP F (1–760 aa), CENP F (761–1130 aa), CENP F (1131–1740 aa), CENP F (1741–2460 aa) and CENP F (2461–2998 aa). mSYCP2 NTD (1–390 aa) was used as the prey in this assay. (C) Western blot analysis of the co-immunoprecipitated mSYCP2 NTR with corresponding constructs from CENP J and F. COS-7 cells were co-transfected using SYCP2 NTD-8x Myc with CENP J (891–1344 aa), CENP J (891–1160 aa), CENP J (1161–1344 aa) and CENP F (2461–2998 aa). (D) The ARLD and SLD of mSYCP2 NTR interacted with CENP J and F proteins in a yeast two-hybrid assay. CENP J (891–1344 aa), CENP J (891–1160 aa), CENP J (1161–1344 aa) and CENP F (2461–2998 aa) were used as bait. The mSYCP2 ARLD and mSYCP2 SLD were used as prey. (E) Western blot analysis of the ARLD and SLD of mSYCP2 NTR co-immunoprecipitated with corresponding constructs from CENP J and F. (F) Proposed model of SC association with the centromere. The N-terminal region of the SYCP1 molecule binds to the central element consisting of SYCE1, SYCE2, SYCE3 and TEX12 proteins. The lateral element is composed of the SYCP1 C-terminal region and the C-terminal regions of SYCP2 and SYCP3. The mSYCP2 NTR is associated with the centromere. Therefore, SYCP2 acts as a bridge to connect the centromere with the SC

To determine the regions required for mSYCP2 NTR binding, we co-expressed several truncations of the above candidates based on protein sequence analysis in a yeast two-hybrid assay. The results indicated that the C-terminal coiled coil region (aa 891–1160) of CENP J is required and sufficient for mSYCP2 NTR binding (Fig. 2A). Compared to CENP J, the full length CENP F showed much weaker interaction with the bait. The C-terminal domain (aa 2,461–2,998) was responsible for interaction with the mSYCP2 NTR (Fig. 2B). Interestingly, it has been proposed that this region interacts with tubulin (Volkov et al., 2015). To validate interactions between mSYCP2 NTR and CENP F or CENP J, we performed co-transfection and immunoprecipitation experiments in COS-7 cells (Lu et al., 2014). Our results were consistent with the yeast hybrid mapping assay (Fig. 2C). Next, we investigated which region of mSYCP2 is the major site of interaction with CENP F or CENP J. Our results revealed that the ARLD, but not the SLD, interacted with CENP J and CENP F (Fig. 2D and 2E). It has been noted that the transfection of full-length CENP J or CENP F genes into COS7 cells resulted in massive cell death. Taken together, our data suggest that the mSYCP2 NTR interacts with the centromere during meiosis I.

Our results, for the first time, provide insight into the possible role of SYCP2 in SC assembly/disassembly. Our structural analysis revealed that mSYCP2 NTR contains two separate subdomains (an ARLD and a SLD) and that the overall conformation appears as a “twisted arm”. The ARLD domain belongs to the armadillo-repeat protein family. Previously published results showed that typical armadillo-repeat units often form a superhelix, which typically provides a platform for many protein partners that transduce Wnt signaling, such as β-catenin. We found that the ARLD of mSYCP2 can also associate with different protein partners, including CENP J and CENP F. We also tried to further address more specific residues of the ARLD that interact with CENP J and CENP F. Three SYCP2-NTR mutants (F166A, F244A and W328A) were built. These mutants were then transfected into COS7 cells and the co-IP experiment was conducted. However, we did not observe any differences between the wild type and mutants in terms of interacting with CENP J and CENP F (Fig. S3). The complex structure of SYCP2 with CENP J or CENP F maybe required to map out the detailed interactions. Interestingly, the SLD domain structurally resembles a Spt16M molecule, which is known as the well-recognized histone protein H2A-H2B; thus, the SLD may be involved in chromatin binding. It was reported that SYCP2 is tightly bound to chromatin in wild type mice (Yang et al., 2006). We speculate that the SLD may contribute to the association of SYCP2 with chromatin. Unfortunately, we failed to observe NTD association with the nucleosome *in vitro*. It is possible that in addition to the SLD, another part of SYCP2 may be required for this association.

The centromere is a multi-protein complex and recruits the kinetochore to form attachments to the microtubules of the mitotic and meiotic spindles. CENP J and CENP F are two components of the centromere. Using yeast two-hybrid screening, we discovered the surprising finding that the SYCP2 NTR associates well with the centromere. It was reported that the C-terminal region of mSYCP2 was found to be responsible for the association of SYCP3 and SYCP1 for SC formation. Consistent with the localization of the SC and the centromere to male MI chromosomes, SYCP2 and SYCP3, as the lateral elements, begin to localize to the chromosome core during the leptotene stage and continue to localize until the diakinesis stage when all but the crossover sites and the centromeres are broken down (Bisig et al., 2012). Therefore, it is reasonable to assume that SYCP2 may act as a bridge that brings the SC and centromere together. SYCP1 has no detectable immunofluorescence signals during the late diplotene stage before the nuclear envelop breaks down; also, parts of SYCP3 begin to gradually disappear during the diplotene stage, whereas the remnants are maintained at the centromere as microtubules for assembly until anaphase I (Bisig et al., 2012). These results indicate that SYCP1 and SYCP3 are less likely to be responsible for the association of the SC with the centromere. Of course, we cannot exclude the possibility that other unidentified proteins may contribute to their association. Thus, we proposed a model in which mSYCP2 bridges the SC with the centromere (Fig. 2F).

In addition, CENP-F displays weak microtubule binding activity on its own; Vergnolle et al. demonstrated that CENP-F provides a link between kinetochores and Ndel1/Nde1/Lis1/Dynein microtubule motor complexes (Vergnolle and Taylor, 2007). We speculate that the residual SYCP2 may act as an indirect mediator between spindle microtubules and the synaptonemal complex, ensuring high-fidelity meiotic chromosome segregation during the MI stage. CENP J proved to be required for both centriole formation and pericentriolar material (PCM) assembly in previous mitosis studies (Hatzopoulos et al., 2013). However, there are no centrioles present during mouse spermatogenesis. We speculate that CENP J may facilitate homologous chromosome segregation. Future experiments on the temporal regulation of these interactions in male meiotic cells are required for a better understanding of the dynamic process.

## Electronic supplementary material

Below is the link to the electronic supplementary material.

Supplementary material 1 (PDF 569 kb)
