# Supplementary material for: Synaptonemal complex protein 2 (SYCP2) mediates the association of the centromere with the synaptonemal complex
Source: Protein Cell. 2017 Feb 1;8(7):538–43. doi: 10.1007/s13238-016-0354-6 (PMC5498334; doi:10.1007/s13238-016-0354-6)
Supplement: Supplementary file 1 — Supplementary material 1 (PDF 569 kb) [file 13238_2016_354_MOESM1_ESM.pdf]

Supplemental Methods

Protein expression and purification

Complementary DNA of SYCP2 (Gene ID: 320558) was obtained from an adult mouse testis cDNA library constructed using a Takara kit and identified by sequencing. The mouse SYCP2 gene fragment (residues 1-390) was cloned into a modified pET-32a vector with an N-terminal Trx fusion protein, His-tag and 3C protease cleavage site and was then transformed into *Escherichia coli* BL21 (DE3) codon plus cells. Overexpression of SYCP2 protein was induced with 0.2 mM isopropyl-b-D-thiogalactopyranoside (IPTG). After growing for 16 h at 25 °C, the cells were collected by centrifugation at 5,000 rpm and homogenized in a buffer containing 50 mM Tris-HCl, pH 8.0; 0.1 mM phenylmethylsulfonyl fluoride (PMSF); 5 mM imidazole; and 500 mM NaCl. After sonication and centrifugation, the supernatant was applied to Ni^{2+} affinity resin (Ni-NTA, Qiagen). The eluted His6-tagged proteins were digested using PreScission protease at 4 °C overnight and were further fractionated using size-exclusion chromatography (Superdex 200, GE Healthcare) with running buffer containing 25 mM Tris-HCl pH 8.0, 250 mM NaCl and 2 mM DTT. The peak fraction was collected and concentrated to ~7 mg/ml for crystallization. The selenomethionine labeled protein SYCP2 was overexpressed in *E. coli* B834 cells with LeMaster media and purified in the same way as the wild type.

Crystallization and data collection

The SYCP2 NTR was crystallized using the hanging-drop vapor-diffusion method at 20 °C by mixing 1 μl of protein with 1 μl of reservoir solution containing 0.1 M Tris-

HCl, pH 6.5 and 25% PEG3350. Stick shaped crystals appeared over two days and grew to full size within one week. Se-Met labeled protein was crystallized similarly. All of the crystals were frozen with a cryoprotectant consisting of the reservoir solution, glycerol increased to 25% and 1 mM DTT.

Native crystal data were collected at the BL-17A beamline of the Photon Factory (Tsukuba, Japan); single-wavelength Se anomalous data were collected for Se-Met-labeled crystals at SSRF beamline BL19U and diffracted to 2.62 Å with a space group of $\boldsymbol{P}2_12_12_1$ and unit cell dimensions of $\boldsymbol{a}$ = 65.68 Å, $\boldsymbol{b}$ = 73.91 and $\boldsymbol{c}$ = 80.64 Å. All datasets were processed and scaled using the HKL2000 software package (Otwinowski and Minor, 1997).

Structure determination and refinement

For SYCP2 NTR structure, 12 Se sites out of a total of 14 were identified by the HKL2MAP program (Pape and Schneider, 2004). The initial electron density map was calculated using PHENIX Autosol (Zwart et al., 2008). The residues were first built automatically using PHENIX Autobuild and then manually built using COOT based on $2\boldsymbol{F}_{obs}$-$\boldsymbol{F}_{calc}$ and $\boldsymbol{F}_{obs}$-$\boldsymbol{F}_{calc}$ Fourier difference maps (Emsley and Cowtan, 2004). After the initial model was built, iterative refinement against the native data set of 2.1 Å was performed using PHENIX Refine and COOT. The orientations of the amino acid side-chains and bound water molecules were modeled on the basis of $2\boldsymbol{F}_{obs}$-$\boldsymbol{F}_{calc}$ and $\boldsymbol{F}_{obs}$-$\boldsymbol{F}_{calc}$ Fourier difference maps. The final structure had an $\boldsymbol{R}_{work}$ of 19.04% and an $\boldsymbol{R}_{free}$

value of 23.89%. Detailed data collection and refinement statistics are summarized in Table S1. The representative electron density maps are shown in Figure S4.

Yeast two-hybrid screening and mapping assay

For yeast two-hybrid screening, mSYCP2 cDNA encoding the N-terminus region (amino acids 1-390) was subcloned into the pGBKT7 vector. The bait strains were cultured by transforming the bait construct into the AH109 yeast strain. A mouse testis cDNA library with 1.3×10^7 colony-forming units (CFU), a recombination rate of 100% and an average insert size of approximately 1.5 kb in the identified library were also transformed into bait strains. Positive transformants were selected on nutrition-restricted plates (SD-Trp-Leu-His-Ade). Prey plasmids were extracted from the candidate clones and then sequenced. To exclude false positive clones, the candidate prey plasmids were retransformed into the AH109 yeast strain along with the bait vector in medium containing 15 mM 3-amino-1,2,4-triazole. Candidate protein domain truncation was also performed using a standard yeast two-hybrid protocol (Clonetech).

Cell culture and transfection

COS-7 (green monkey kidney) cells were cultured at 37 °C in 5% (v/v) CO_2 in air in Dulbecco's modified Eagle's medium (DMEM, Sigma) with 10% fetal bovine serum (FBS, PAN, Germany).

Gene fragments corresponding to CENP J (891-1344 aa), CENP J (1161–1344 aa), CENP J (891-1160 aa) and CENP F (2461–2998 aa) were cloned into the pEGFP-C1 vector (Clonetech). The genes of mSYCP2 NTR (1-390 aa), ARLD (1-270 aa) and SLD (276-390 aa) were also cloned into the modified pcDNA4/myc-His A vector

(Invitrogen) containing 8 X Myc tags and confirmed by sequencing. Cells were transfected at 80% confluency with 6 μg of DNA using PEI according to the manufacturer's instructions and were used after 24 h for co-immunoprecipitation.

Co-immunoprecipitation and Western blotting

Transfected COS-7 cells were collected in general PBS buffer and washed three times. Cells harvested from a 10 cm culture dish were lysed in 500 μl of lysis buffer (1% Triton X-100, 0.5% deoxycholate, 0.1% SDS, 1 mM β-glycerolphosphate, 1 mM Na_3VO_4, 1 mM EDTA, 1 mM EGTA, 1x PBS and Roche protease inhibitor cocktail, adding PMSF for final concentration to 1 mM before use). Then, after incubation on ice for 1 h, the lysate was centrifuged at 13,000 ×*g* for 40 min. A total of 40 μl of 1:1 protein A/G agarose (Pierce) slurry was incubated with 2 mg of anti-Myc antibody for 1 h and then washed twice with cold lysis buffer. The cell extract supernatant was added to antibody-coupled protein A/G beads and incubated for 3 h at 4 °C. The beads were washed three times for 10 min with cold lysis buffer, followed by elution with 40 μl of 2X SDS-loading buffer. Subsequently, 10 μl of the elute was loaded onto an 11% SDS-PAGE gel and transferred onto a PVDF membrane for Western blot analysis. The immunoblot analysis was conducted using a primary antibody against GFP. Protein-antibody complexes were detected using enhanced chemiluminescence.

Circular dichroism spectroscopy. Protein secondary structure was recorded in a MOS-450 CD spectrometer (BioLogic) equipped with a temperature controller. Spectral data were measurement at 22℃ using a 1-mm path length quartz cell with 10 μM protein within the wavelength of 190-260 nm.

Accession Numbers

Atomic coordinates and structure factors have been deposited in the Protein Data Bank with accession numbers 5IWZ.

References

Emsley, P., and Cowtan, K. (2004). Coot: model-building tools for molecular graphics. Acta Crystallogr. D Biol. Crystallogr. 60, 2126-2132.

Otwinowski, Z., and Minor, W. (1997). Processing of X-ray Diffraction Data Collected in Oscillation Mode. Method. Enzymol. 276, 307-326.

Pape, T., and Schneider, T.R. (2004). HKL2MAP: a graphical user interface for macromolecular phasing with SHELX programs. J. Appl. Crystallogr. 37, 843-844.

Zwart, P.H., Afonine, P.V., Grosse-Kunstleve, R.W., Hung, L.W., Ioerger, T.R., McCoy, A.J., McKee, E., Moriarty, N.W., Read, R.J., Sacchettini, J.C., *et al.* (2008). Automated structure solution with the PHENIX suite. Method. Mol. Biol. 426, 419-435.

Table S1 Data collection and refinement statistics of mSYCP2 NTD

Data collection		
Crystal name	**Wild-type**	**Se-Met-crystal**
Space group	$P2_12_12_1$	$P2_12_12_1$
a,b,c (Å)	65.78, 74.68, 80.51	65.68, 73.91, 80.64
Molecule/asu	1	1
Wavelength (Å)	0.9791	0.9789
Resolution range (Å)	50-2.10	50-2.62
	(2.14-2.10)	(2.71-2.62)
No. of unique reflections	23,639	12,291
Redundancy	7.2(7.4)[a]	14.3(14.7)[a]
R_{sym} (%)[b]	7.9(51.7)[a]	9.3(26.4)[a]
I/σ	21.7(5.5)[a]	36.9(9.9)[a]
Completeness (%)	98.9(98.7)[a]	99.9(99.7)[a]
FOM	---	0.484/0.721(after DM)
Refinement		
Resolution range (Å)	32.47~2.10 (2.18-2.10)	
$R_{crystal}$ (%)[c]	19.04	
R_{free} (%)[d]	23.89	
$RMSD_{bond}$ (Å)	0.008	
$RMSD_{angle}$(°)	1.475	
Number of		
solvent atoms	109	
Residues in (%)		
most favored	94.2	
additional allowed	5.8	
generously allowed	0	
disallowed	0	
Average B factor of		
protein	42.11	
Solvent	42.43	

[a] the highest resolution shell.

[b] $R_{sym} = \sum_j \left| \langle I \rangle - I_j \right| / \sum \langle I \rangle$

[c] $R_{crystal} = \sum_{hkl} \left| F_{obs} - F_{calc} \right| / \sum_{hkl} F_{obs}$

[d] R_{free}, calculated the same as $R_{crystal}$, but from a test set containing 5% of data excluded from the refinement calculation

Supplementary Figure 1

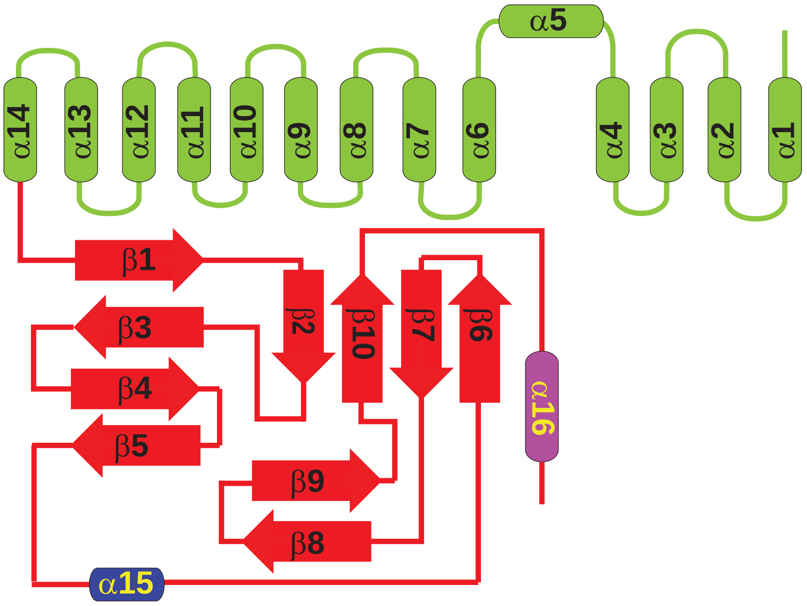

Secondary structure diagram of the mSYCP2 NTR.

Supplemental Figure 2

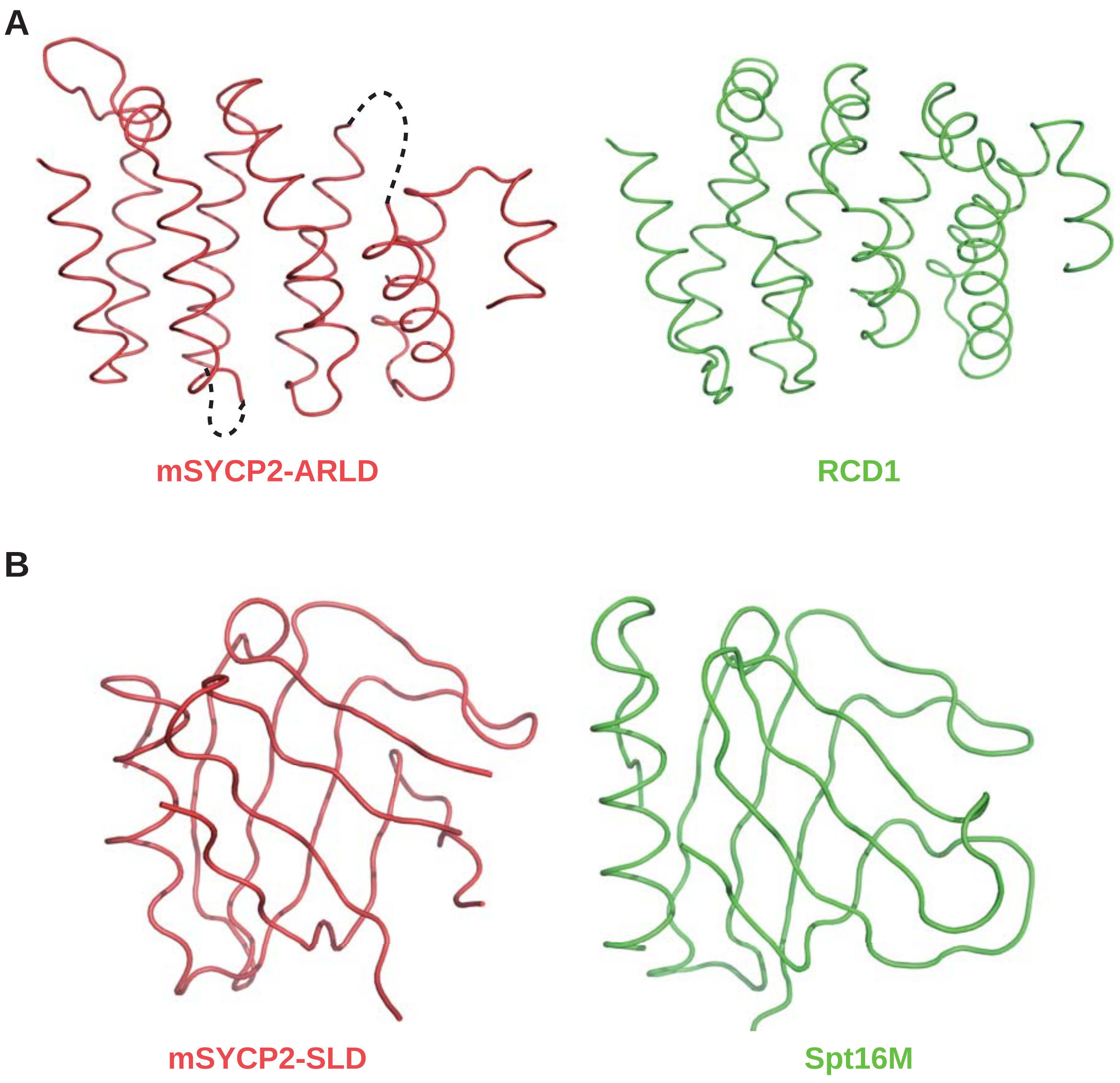

Side-by-side structural comparison of mSYCP2-NTR with RCD-1 (green, PDB ID:2FV2) and Spt16M (green, PDB ID: 4KHA)

Supplemental Figure 3

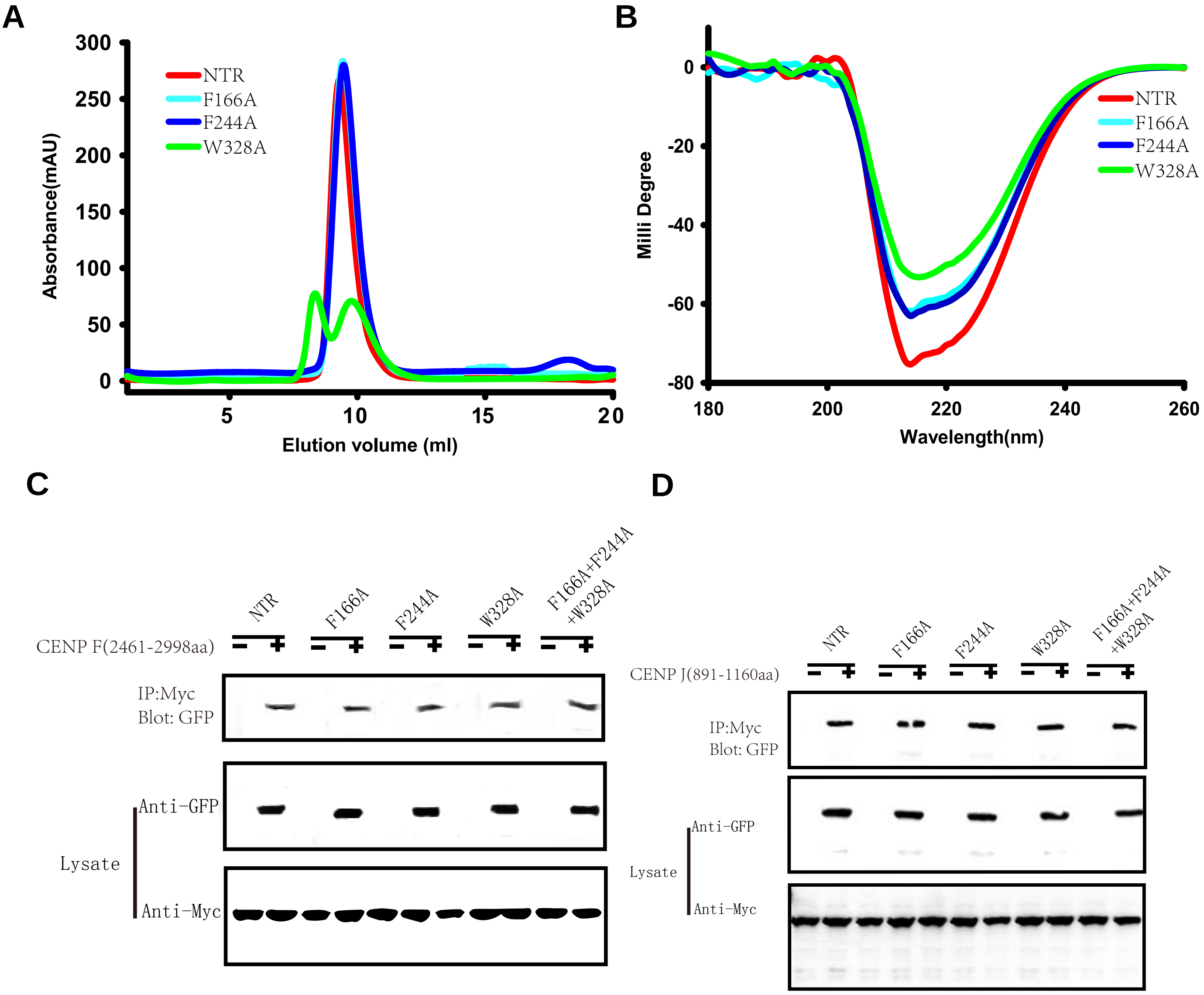

Mutational analysis of SYCP2-NTR with CENP F and CENP J. (**A**) Gel-filtration chromatography analysis of the purified wild type and mutants. (**B**) Secondary structure analysis profile of wild type and mutants by circular dichroism. Western blot analysis of the CENP F (**C**) or CENP J (**D**) co-immunoprecipitated with the wild type and mutants.

Supplemental Figure 4

A

B

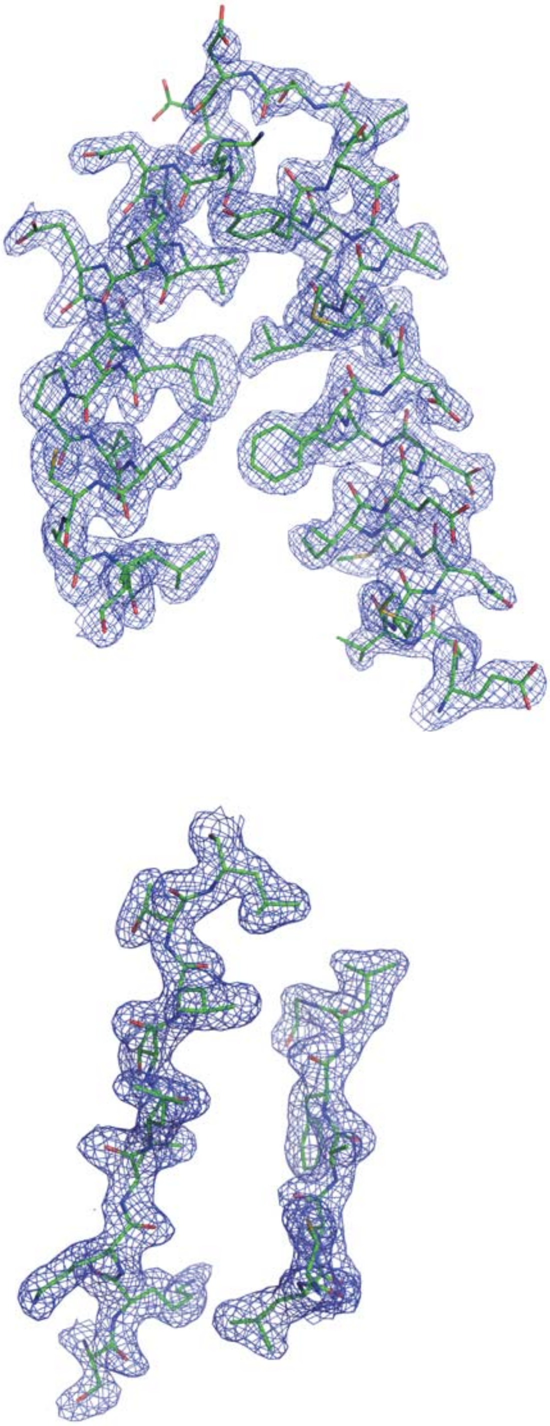

The representive electron density map of mSYCP2-NTR structure. The map was contoured at 1.0 σ and shown as blue mesh. (A) The map derived from initial phases. (B) The map after refinement.
